# Isolating perceptual conflict: a theoretical and methodological analysis of the Task-Irrelevant Paradigm

**DOI:** 10.3389/fpsyg.2026.1603617

**Published:** 2026-05-04

**Authors:** Yian Guo, Yanan Wu, Yuanqing Yao, Ke You, Guangping Zhao

**Affiliations:** 1 School of Education and Psychology, Minnan Normal University, Zhangzhou, China; 2 Quanzhou Huaguang Vocational College, Quanzhou, China; 3 Institute of Applied Psychology, Minnan Normal University, Zhangzhou, China; 4 Fujian Province Key Laboratory of Applied Cognition and Personality, Zhangzhou, China; 5 Suqian Zeda Vocational and Technical College, Suqian, China; 6 Anhui Finance and Trade Vocational College, Hefei, China

**Keywords:** perceptual conflict, task-irrelevant paradigm, stroop effect, cognitive control, conflict interference paradigm

## Abstract

The conflict interference effect, exemplified by the Stroop effect, serves as a key indicator for studying conflict processing and cognitive control and is typically categorized into two conflict components within conflict classification theories. Perceptual conflict is constrained by task settings in typical paradigms, making precise detection challenging. This article reviews the literature on perceptual conflict, proposes the Task-Irrelevant Paradigm (TIP) as an experimental strategy to independently investigate perceptual conflict, and provides a more systematic analysis of its theoretical, empirical, and cognitive-processing foundations. The TIP offers an effective method for more deeply exploring perceptual conflict, and a clearer framework for understanding how such conflict may persist under task-irrelevant conditions, demonstrating broad applicability across conflict interference paradigms and potential for studying implicit associations. Current empirical research involving the TIP is still relatively limited, and future studies should focus on refining task designs, clarifying boundary conditions, and advancing multimodal integration research.

## 1 Introduction

The Stroop paradigm (Stroop, 1935) is a widely used research framework in cognitive psychology (MacLeod, 1991). Its core principle is that when the semantic content of words conflicts with their displayed color, response times become longer and error rates in color recognition increase, producing the so-called Stroop effect. Experiments based on the Stroop paradigm have expanded beyond the original color-word recognition task to include various forms of cognitive conflict involving space, emotion, shape, numbers, and phonemes (Bush et al., 2006; Freitas et al., 2023; Ke et al., 2020; Kinoshita and Verdonschot, 2021; Sun et al., 2023; Van Hooff et al., 2008; Viviani et al., 2024; Xiao et al., 2016). By varying specific task features, researchers can investigate different types of cognitive interference and control mechanisms.

The Stroop effect has had a substantial impact on cognitive psychology and related fields through its integration with multiple research techniques. For example, functional magnetic resonance imaging (fMRI) has identified brain regions activated during Stroop tasks (Bush et al., 1998; Fang et al., 2022; Grandjean et al., 2012; van Veen and Carter, 2005; Verdolini et al., 2023). Event-related potentials (ERP) have clarified the temporal dynamics of cognitive conflict processing (Cardoso et al., 2024; Fang et al., 2022; Liotti et al., 2000). Eye-tracking technology has deepened understanding of how individuals allocate attention while handling cognitive conflict (Rizzo et al., 2022; Vakil et al., 2019), and virtual reality has improved the ecological validity of Stroop experiments (Asbee et al., 2022; Pyne et al., 2023). Together, these methods have advanced the study of cognitive mechanisms and extended the use of the Stroop effect to fields such as neuroscience, social psychology, educational psychology, and industrial and organizational psychology (Banich, 2019; Huang et al., 2020; Henschel et al., 2021; Panta et al., 2020; Shi and Yu, 2022; Siegel et al., 2022). They have also broadened the methodological basis for interdisciplinary research.

In cognitive psychology, the mechanisms underlying the Stroop effect reveal the interaction between automatic and controlled processing in cognitive tasks (Liu et al., 2015). For example, analyzing how participants suppress reading in order to identify colors in Stroop tasks can deepen our understanding of cognitive control mechanisms (Bianco et al., 2021; Scarpina and Tagini, 2017). This, in turn, supports the use of the Stroop paradigm in assessing and training cognitive flexibility and control functions. In clinical psychology, adjusting the complexity of Stroop tasks can help assess executive function impairments in neurodevelopmental disorders and monitor therapeutic effects and cognitive recovery processes (Alders et al., 2019; MacLeod and MacDonald, 2000; Raz et al., 2007). The Stroop effect also helps explain how people allocate attention and manage resources when processing conflicting information (Parris et al., 2022), which in turn informs the design of information-processing systems and decision-making tools for high-pressure environments such as air traffic control and emergency response. Thus, exploring the mechanisms of the Stroop effect not only advances theoretical development but also strengthens the link between basic science and practical applications (Feng et al., 2018).

The Stroop effect is commonly understood as a conflict effect arising from different cognitive processes, including automatic and deliberative processes. Within dual-process theory, automatic processes generally refer to processing that can be initiated rapidly by relevant stimulus cues, unfolds relatively autonomously, and depends less on attention and the maintenance of working memory. Deliberative processes, by contrast, rely more on the allocation of attention, rule selection, and task-goal control (Schneider and Shiffrin, 1977; Evans and Stanovich, 2013). From this perspective, the mechanism of the Stroop effect can be explained as follows: interfering information in the stimulus is automatically activated and enters processing, whereas task performance requires the individual to maintain a processing direction consistent with the current task goal. When these two are inconsistent, conflict interference arises, leading to longer response times or increased errors (Cohen et al., 1990; Littman et al., 2019; MacLeod, 1991). The Stroop effect can therefore also be viewed as a typical conflict interference phenomenon that emerges when automatic processing competes with deliberative control.

Although the Stroop paradigm has primarily been used as a key tool for investigating conflict processing in individuals, mainly through interference-related response delays observed during experimental tasks, and although the behavioral interference reflected in the Stroop effect has been widely accepted as an effective indicator for explaining conflict processing, this single-effect interpretation has certain limitations. In particular, it does not always fully reveal the mechanisms involved across the whole cognitive process, especially when different types of conflict need to be distinguished.

In addition, a growing number of studies have shown that, beyond the Stroop effect, other similar conflict interference effects are also important. Although these paradigms differ in their task forms, they share a common focus on the processing and control of conflicting information and, in essence, reflect cognitive mechanisms similar to those underlying the Stroop effect.

We reviewed three major theories for classifying conflict interference effects and, drawing on previous studies that have decomposed conflict components from the perspectives of task structure, stimulus properties, processing pathways, and response selection, summarized the distinctive features of perceptual conflict and its research limitations. Furthermore, we proposed a novel experimental paradigm—the Task-Irrelevant Paradigm—aimed at enabling a more in-depth and fine-grained analysis of the Stroop effect and related conflict interference effects. We also analyze this paradigm in detail to support its further theoretical development and broader application.

### 1.1 Informational conflict and task conflict

Goldfarb and Henik (2007) proposed that the Stroop task involves two distinct types of conflict. The first is informational conflict between the incongruent word and color. This conflict arises from a mismatch between task-relevant and task-irrelevant information (e.g., “blue” and “red”) and is manifested in incongruent trials. The second is task conflict between the relevant color-naming task and the irrelevant, stimulus-driven word-reading task. This conflict is present in both incongruent and congruent trials because the word tends to trigger an automatic reading response (Littman et al., 2019).

Task conflict is considered one of the core features of the Stroop task and other conflict interference paradigms, such as the Simon task, Flanker task, and global–local paradigm (Lee and Sewell, 2024; Littman et al., 2019; Parris, 2014). Unlike informational conflict, task conflict does not depend on semantic incongruence. Instead, it arises from the parallel activation of two competing task sets. This framework distinguishes different levels of interference processing, namely the task-set level and the semantic level, and also highlights the role of cognitive control. For example, Kalanthroff et al. (2013, 2018) and Kalanthroff and Henik (2014) further examined factors that influence task conflict. They proposed that when individuals have strong proactive control, they can rapidly suppress the activation of non-target task sets at an early stage of task presentation, thereby substantially reducing or even eliminating task conflict. By contrast, when control resources are weakened, such as under distraction, fatigue, or task-switching conditions, task conflict becomes more pronounced and may even impair performance in congruent trials. This pattern has been described as the “reverse facilitation” effect.

The distinction between informational conflict and task conflict is grounded in the structure of the experimental design. These two types of conflict are not treated as incidental byproducts of subjective processing. Rather, they are regarded as inherent features of the Stroop paradigm and related cognitive interference paradigms at the levels of stimulus configuration and task setup. Specifically, informational conflict arises from the presence of two dimensions within the stimulus materials themselves, whereas task conflict stems from the dual-task competitive structure embedded in the experimental task. In this structure, color naming is explicitly activated as the target task, whereas word reading is unconsciously triggered as the non-target task because of its high degree of automaticity. This dual structure of task and stimulus dimensions allows multiple processing channels to be activated automatically within the cognitive system, even when participants are not instructed to process word meaning, thereby producing conflict. In other words, this account suggests that the two types of conflict in the Stroop task, as well as in similar nonverbal interference tasks, reflect internal features of the experimental design and represent the sources of conflict that individuals must resolve during task execution.

### 1.2 Perceptual conflict and response conflict

From the perspective of individual cognitive processing, the components of the Stroop effect are generally interpreted in two ways: perceptual conflict, which occurs at the stage of perceptual identification, and response conflict, which arises during response selection (Besner and Stolz, 1999; Hershman and Henik, 2020; Li et al., 2013; MacLeod, 1991; Parris et al., 2019; Parris et al., 2022; Quétard et al., 2023; Steinhauser and Hübner, 2009; Szűcs and Soltész, 2010). The perceptual conflict hypothesis suggests that when the semantic meaning and the printed color of a word are incongruent, individuals detect a mismatch at the level of the overall stimulus, which gives rise to conflict. Because cognitive resources available for conflict processing are limited, this type of conflict places additional demands on the information-processing system and therefore requires extra processing time (Doehrman et al., 1978; Hershman and Henik, 2019). In contrast, the response conflict hypothesis holds that, during response selection, the incongruent word and color generate competing response tendencies. Both tendencies attempt to access the same response channel, ultimately producing delayed responses (Doehrman et al., 1978; Steinhauser and Hübner, 2009).

The core idea of the perceptual conflict hypothesis is that the time required for recognition or encoding differs between incongruent and congruent conditions. The brain needs more time and cognitive resources to process inconsistent input information (Hock and Egeth, 1970; Scerrati et al., 2017; Shichel and Tzelgov, 2018). As a result, the Stroop effect emerges, and the resulting interference or attentional competition is viewed as a consequence of increased encoding time rather than its cause. By contrast, the response conflict hypothesis explains the cognitive interference observed in the Stroop task mainly in terms of response selection. It proposes that the Stroop effect is primarily caused by response competition during the execution stage (Zhao et al., 2015). When individuals are presented with stimuli in which word meaning and font color are incongruent, the reading response is activated first (Augustinova et al., 2017; MacLeod and MacDonald, 2000). As a result, irrelevant word information reaches the stage of response initiation before the relevant color information, thereby producing response delay and interference (Doehrman et al., 1978).

The response conflict account emphasizes interference during response selection and focuses mainly on the later stages of decision-making and execution (Atkinson et al., 2003). However, it often does not fully consider earlier phases of task processing, such as the automatic and unconscious influences involved in perception and attention allocation. In other words, this account treats the Stroop effect mainly as a problem of executive control, with less emphasis on earlier stages of information processing. Studies have shown that automatic processing at the perceptual stage plays a crucial role in the generation and resolution of conflict. For example, early perceptual conflict can trigger automatic attention allocation (Cohen et al., 1990). Although the perceptual conflict account offers a perspective centered on the holistic processing of conflicting information, current research still relies mainly on the traditional Stroop paradigm, which does not effectively distinguish the respective contributions of these two types of conflict to the observed experimental effects (Scerrati et al., 2017). This methodological limitation may oversimplify interpretations based on perceptual conflict and, in turn, constrain a more comprehensive understanding of the mechanisms underlying conflict interference effects (Chmielewski and Beste, 2019). For example, some studies using modified versions of the classic paradigm found ERP evidence for perceptual conflict without corresponding behavioral effects (Cheng et al., 2021; Li et al., 2013).

### 1.3 Stimulus–stimulus conflict and stimulus–response conflict

Dimensional Overlap Theory posits that when stimuli and responses overlap across multiple dimensions, perceptual processing becomes more complex, thereby reducing both processing speed and accuracy (Kornblum, 1994; Kornblum et al., 1990; Kornblum et al., 1999; Liu et al., 2010). According to this theory, conflict effects observed in stimulus–response compatibility paradigms arise from two sources: stimulus–stimulus conflict (SSC), which refers to conflict between relevant and irrelevant stimulus dimensions, and stimulus–response conflict (SRC), which refers to conflict between an irrelevant stimulus dimension and the relevant response dimension (Cheng et al., 2021).

Within this framework, “dimensional overlap” does not refer to cognitive processing across all stimulus and response dimensions. Rather, it refers specifically to structural overlap along task-relevant dimensions. In other words, both SSC and SRC stem from structural interference at the level of task dimensions. More specifically, SSC arises from a contradiction between two dimensions within a stimulus: one is the task-relevant dimension that must be processed to complete the task, and the other is a task-irrelevant but automatically activated dimension. SSC therefore interferes with recognition and processing of the target stimulus on the task-relevant dimension through incongruent information from the irrelevant dimension, leading to delayed responses. For example, in a color-word Stroop task, color is the task-relevant dimension, whereas word meaning, although not task-relevant, overlaps with color at the semantic level and is extracted automatically by the cognitive system, thereby disrupting color processing and delaying responses. By contrast, SRC is characterized by an incompatible mapping between information from a task-irrelevant dimension and the target response. That is, the irrelevant information automatically activates an incorrect response tendency and directly interferes with selection and execution of the correct response. For instance, in the Simon task, participants are instructed to respond with left or right keypresses on the basis of color, whereas stimulus location (left or right) triggers a spatially corresponding response tendency, thereby producing SRC.

Although Dimensional Overlap Theory clearly identifies SSC and SRC as two mechanisms underlying interference effects, some studies have reported cases in which its theoretical predictions are not supported (Cheng et al., 2021; Paap et al., 2020). This suggests that the theory may overlook certain perceptual conflicts that arise from individuals’ sensitivity to structural incongruence within the stimulus itself, even when such conflict is unrelated to the current task goal and does not activate a specific response tendency. Algom and Chajut (2019) argued that when individuals are presented with multidimensional stimuli, they form a holistic perceptual experience of informational consistency or coherence. If the stimulus contains “structural disharmony,” this can be detected at early stages of perceptual processing. Although such conflict may involve only limited engagement of higher-level cognitive control, it still consumes processing resources and may lead to slower processing or shifts of attention. Because this type of conflict does not directly affect completion of the task goal, it does not require the brain to suppress interfering information or enhance processing of goal-relevant attributes through dynamic adjustments in information-processing strategy. Its influence on decision-making and behavioral execution may therefore be relatively limited (Jha and Kiyonaga, 2010; Surrey et al., 2017).

### 1.4 Current theoretical analysis

A systematic review of the theoretical development of conflict interference paradigms shows that current mainstream theories tend to classify conflict into two functionally distinct components.

The informational conflict and task conflict framework focuses on the source composition of conflict effects. It emphasizes that task conflict reflects structural competition between different task sets embedded in the experimental paradigm and is regarded as a fundamental feature shared by most conflict tasks. Informational conflict, by contrast, reflects interference caused by incongruent information within the stimulus itself during the processing of goal-relevant attributes. The perceptual conflict and response conflict framework, in contrast, adopts a processing-course perspective and locates conflict at two distinct stages of cognitive processing. Perceptual conflict primarily arises at an early stage of information integration and originates from disruption of overall stimulus coordination, whereas response conflict emerges at the later stage of response selection and is characterized by direct competition between alternative response tendencies. Dimensional Overlap Theory emphasizes the structural conditions under which conflict arises. By distinguishing between SSC and SRC, it proposes that interference effects depend on how task-relevant and task-irrelevant dimensions overlap during encoding and how conflicting mappings are structurally organized, thereby providing a structural mapping account of the generation of conflict interference.

Among the conflict mechanisms discussed above, perceptual conflict has a distinctive feature: it does not depend on explicit activation of the task-relevant dimension, nor does it necessarily trigger overt response competition, yet it can still produce interference effects at early stages of processing. In this sense, perceptual conflict can be regarded as a form of implicit conflict that is neither response-driven nor directly dependent on response mapping.

However, in most nonverbal interference tasks, perceptual conflict is difficult to isolate cleanly from other types of conflict effects (Paap et al., 2020). This ambiguity stems from the structural characteristics of typical conflict interference paradigms, in which the source of perceptual conflict often overlaps with the task goal, making it highly likely to also induce response conflict. Although some neuroimaging studies suggest that perceptual conflict may exert independent effects at early stages of brain processing, the structure of experimental tasks often prevents this type of conflict from being effectively isolated. This limitation is especially important in studies of implicit cognitive interference, where certain task information may implicitly cue participants to possible relations among experimental variables and thus become more susceptible to proactive control.

We identified a group of studies that adopted task-irrelevant designs (Hatukai and Algom, 2017; Li et al., 2013; Oren et al., 2023; Wolska, 2021; Yuanqing et al., 2024; Zhao and Wang, 2013), which appears to provide an effective approach for examining perceptual conflict. Building on this line of work, we propose the concept of the Task-Irrelevant Paradigm and aim to clarify its theoretical basis, operational characteristics, potential applications, and limitations in conflict-processing research.

## 2 Task-irrelevant paradigm

Task-Irrelevant Paradigm (TIP) refers to an approach derived from classic conflict interference paradigms, in which the original task requirement is replaced with a task unrelated to the conceptual dimensions of informational conflict and task conflict. For example, in the Stroop paradigm, the original stimulus conflict is retained, such as the word “RED” printed in blue. However, a new task goal is introduced that is unrelated to the conflicting dimensions, such as judging brightness, size, or orientation. Compared with typical paradigms, the key advantage of the Task-Irrelevant Paradigm is that it allows participants to complete the task without suppressing interfering information, thereby eliminating the expression of both task conflict and informational conflict. What remains is the influence of perceptual conflict, arising from the inherent properties of the stimulus, on task performance.

### 2.1 Theoretical development

The task-irrelevant design within the Stroop paradigm can be traced back to Bekçi and Karakaş (2009). Based on the typical Stroop paradigm, they introduced a new experimental task: judging whether the color and semantic meaning of a word were consistent. This task replaced the traditional requirement to identify either the color or the word meaning of the stimulus. Their behavioral findings showed that perceptual conflict could significantly affect task performance even in the absence of response conflict. Although the task used in their experiment did not strictly qualify as a “task-irrelevant” one, the design reduced the association between Stroop conflict and the task goal. It therefore provided a preliminary theoretical foundation for the development of the Task-Irrelevant Paradigm within the Stroop framework.

Li et al. (2013) conducted a comparative study using both the standard Task-Irrelevant Paradigm and the typical Stroop paradigm to investigate the behavioral and cognitive-neural manifestations of perceptual conflict. In the task-irrelevant condition, participants were asked to judge the rotational status of the word stimuli, specifically whether each word was upright or rotated. This design completely shifted the task focus away from the traditional color–semantic conflict and toward an unrelated rotation judgment, thereby eliminating response conflict more thoroughly. However, the task-irrelevant condition in this study did not yield behavioral evidence of a conflict effect. Zhao and Wang (2013) proposed an alternative task-irrelevant design within the Stroop framework. They embedded Stroop stimuli within geometric figure outlines and treated the Stroop conflict information as a task-irrelevant background stimulus. This design was intended to examine whether perceptual conflict could still elicit a congruency sequence effect under conditions entirely devoid of response conflict. The results showed that even when Stroop stimuli were completely task-irrelevant, participants still exhibited typical congruency sequence effects across successive trials.

Hatukai and Algom (2017) demonstrated the broad extensibility and application potential of the Task-Irrelevant Paradigm in cognitive research and suggested that this design framework deserves greater theoretical and empirical attention. Unlike other conflict interference studies that focus on the effects of conflict between variables, this study was the first to extend the analytical perspective systematically to the influence of Stroop stimuli on task-irrelevant processing. The researchers adopted various forms of task-irrelevant judgments, such as vertical position, font width, and image contours, to match different types of Stroop stimuli. The results showed that even when the conflicting stimuli were entirely irrelevant to the task, they still influenced cognitive processing.

Oren et al. (2023) pointed out that in many variants of the Stroop paradigm, the supposedly “task-irrelevant” attributes often still belong to the same conceptual dimension as the target attribute (e.g., color, emotion, or semantics), meaning that they are not truly task-irrelevant. To break the dimensional coupling between task and conflict attributes, their study introduced an animate/inanimate judgment task for Stroop stimuli involving colors, pictures, and words. More importantly, this study represents a critical step toward a precise definition of the Task-Irrelevant Paradigm. The authors defined “task-irrelevant” as referring to an attribute from a different conceptual dimension than the task. This definition provides an important foundation for the development and application of the paradigm.

The Task-Irrelevant Paradigm has attracted attention not only in Stroop-like research but also in studies involving other classic conflict interference paradigms. For instance, in the typical Navon task, participants are required to identify compound stimuli composed of both local and global information, with the task goal usually focusing on one of the dimensions involved in the conflict. In one study, however, participants were asked to judge the brightness of Navon stimuli, a task unrelated to the conflicting dimensions (Wolska, 2021). The results showed that, under certain parameter settings, the perceptual conflict generated by Navon stimuli could still produce interference effects. Another study adopted a similar logic and required participants to judge the orientation (up or down) of the local elements within Navon stimuli, thereby creating a task-irrelevant condition (Yuanqing et al., 2024).^1^ The findings demonstrated that perceptual conflict can generate robust interference effects within the Task-Irrelevant Paradigm and that these effects are supported by cognitive-neural evidence.

The integration of the Task-Irrelevant Paradigm with the Flanker task has not yet received focused attention. Only a few studies have experimented with task-irrelevant designs in this context, and a standardized Task-Irrelevant Paradigm has not yet been established. For example, in one study based on the typical Flanker paradigm, researchers inserted a surprise task to examine whether a stimulus unrelated to the conflict task could activate a certain level of attentional processing (Buetti et al., 2014). They emphasized that the so-called “task-irrelevant distractors” in the typical Flanker task are actually attentionally relevant, and that traditional interpretations have mistakenly treated them as task-unrelated interference. Furthermore, in another study, a task-irrelevant stimulus was inserted after the Flanker task to explore how the execution of a conflict task might influence the processing of unrelated information (Ligeza and Wyczesany, 2017). The results showed that, under certain conditions, such task-irrelevant stimuli could trigger attentional processing in the brain.

### 2.2 Characteristics of the task-irrelevant paradigm

Existing research suggests that the Task-Irrelevant Paradigm provides an effective approach for investigating the role of perceptual conflict in conflict interference paradigms, with researchers showing greater interest in the Stroop effect than in other conflict effects. Parris et al. (2022) criticized current research methods, namely typical paradigms, and argued that behavioral studies often fail to isolate the specific stage at which interference occurs. The Task-Irrelevant Paradigm allows perceptual conflict to be examined independently. However, empirical research on this paradigm remains limited, and its design requirements and methodological details still need further refinement and standardization. For example, different task-irrelevant conditions may differ in their sensitivity to perceptual conflict. Examining these differences could help refine experimental parameters and improve understanding and application of the paradigm. This section systematically analyzes the characteristics of the Task-Irrelevant Paradigm and aims to extend the theoretical discussion of the mechanisms underlying perceptual conflict in the Stroop effect and related conflict phenomena.

#### 2.2.1 A more isolated perceptual conflict effect

In typical conflict interference paradigms, perceptual conflict and response conflict often co-occur, making them difficult to distinguish at the behavioral level. To decompose this compound effect, previous studies have typically adopted two design approaches.

The first approach induces interference between stimulus attributes within the same dimension while controlling response-level irrelevance (e.g., Cheng et al., 2021; Paap et al., 2020). Specifically, in Paap et al. (2020), one condition presented an upward-pointing arrow at the bottom of the screen. Although the arrow’s direction had no direct mapping to the response keys, the inconsistency between its visual orientation (“up”) and its actual spatial position (“down”) created a cognitive mismatch, thereby inducing perceptual conflict between stimulus features.

The second approach increases the number of experimental conditions so that some stimuli that trigger perceptual conflict are mapped to the same response key (e.g., Chen et al., 2013; Hershman et al., 2024). For example, Chen et al. (2013) used a classic 4:2 mapping design in both Stroop and Flanker tasks. This method relied on specific stimulus–response pairings to separate the two types of interference behaviorally. In this design, four combinations of semantic and color information were used, with every two color categories mapped to one response key (e.g., red and yellow to the left key; blue and green to the right key).

Although both approaches can eliminate or reduce the influence of response conflict to some extent, they still have notable theoretical and methodological limitations. The first method, although it does not directly induce response conflict, often includes “task-irrelevant” interfering information that remains conceptually associated with opposing response tendencies. In addition, because informational conflict overlaps dimensionally with the task goal, task conflict and response conflict cannot be completely ruled out (Parris, 2014). The second method attempts to eliminate response conflict through shared response keys, but it relies heavily on participants’ comprehension and retention of complex stimulus–response rules. Participants must learn and apply multiple color–response or semantic–response pairings. Under these conditions, even if stimuli such as “red” and “yellow” are mapped to the same response key, they may still follow independent pairing pathways (Hommel et al., 2001; Kornblum et al., 1990). If the specific response-mapping channels activated by each stimulus are inconsistent, then conflict, competition, or delayed processing may still arise during execution, even when the final keypress is the same (Hasshim and Parris, 2015; Proctor and Vu, 2006).

In contrast, the Task-Irrelevant Paradigm separates processing pathways and conceptually decouples task goals from conflict dimensions by assigning the task to a perceptual attribute that is entirely unrelated to the conflict information, such as brightness, orientation, or font width. Participants do not need to consciously process the conflicting attribute, which remains outside the task focus. Accordingly, any influence that still emerges can be attributed only to automatic activation and unintentional interference at the perceptual level. Oren et al. (2023) provided empirical support for this inference through a Stroop-like Task-Irrelevant Paradigm. Their behavioral results showed that conflict interference still occurred under task-irrelevant conditions. More critically, fMRI findings indicated that this interference effect was primarily associated with activity in the intraparietal sulcus (IPS), rather than in the prefrontal control regions commonly observed in task-relevant conflict processing in typical paradigms.

Therefore, the Task-Irrelevant Paradigm not only avoids the complexity of response mapping and the elicitation of response tendencies at the operational level, but also provides a structurally grounded way to isolate perceptual conflict. This makes it possible to detect the intrinsic characteristics of perceptual conflict under conditions of minimal strategic interference and non-task-related processing. This feature has both theoretical and practical value. For example, in intervention domains such as psychotherapy and cognitive training, accurately identifying different types of cognitive conflict is essential for designing effective strategies (Shichel and Tzelgov, 2018). Without precise differentiation among conflict types, the interventions developed may fail to address the actual problems individuals face, thereby limiting their effectiveness.

#### 2.2.2 Sustained effect of perceptual conflict

Several Stroop studies using neuroimaging techniques have shown that, under incongruent conditions, stimulus materials can elicit both early perceptual ERP components and later response-related components (Boenke et al., 2009; Li et al., 2013). These components are associated with attentional selection of visual information as well as conflict monitoring and control (Haciahmet et al., 2023; Henkin et al., 2010; Simon and Berbaum, 1990; West and Alain, 1999; Weibin et al., 2023; Yan et al., 2021). At the same time, the Stroop effect has been found to increase activity in brain regions involved in perceptual processing, such as the visual cortex, suggesting that the initial analysis and integration of visual features may already involve conflict processing (Kamitani and Tong, 2005; Rauss et al., 2011). However, methodological limitations and theoretical oversimplifications have constrained our understanding of the mechanisms underlying conflict effects (Chmielewski and Beste, 2019). For example, research on spatial Stroop tasks has shown that the spatial configuration of a task can significantly affect processing speed and accuracy, indicating that spatial and other dimensions need to be considered when interpreting the Stroop effect (MacLeod and MacDonald, 2000). Therefore, it is not appropriate to attribute conflict effects observed at two different stages directly to two distinct types of conflict. In particular, in typical paradigms, response conflict during the response-selection stage often dominates the experimental outcome, making it difficult to determine whether perceptual conflict is also involved.

The Task-Irrelevant Paradigm provides both methodological and empirical support for a more complete investigation of the processing mechanisms of perceptual conflict. First, Task-Irrelevant Paradigm studies combined with ERP techniques have consistently detected the N450 component associated with conflict detection and the Late Positive Complex (LPC) associated with conflict resolution (Li et al., 2013; Yuanqing et al., 2024). These findings indicate that even when perceptual conflict is irrelevant to the task, it still recruits neural resources during the mid-to-late stages of processing, reflecting its sustained influence and regulatory role in cognitive processing. Second, two Flanker studies using designs approximating the Task-Irrelevant Paradigm showed that even stimulus information unrelated to task goals could still trigger a certain degree of attention and processing (Buetti et al., 2014; Ligeza and Wyczesany, 2017). Ligeza and Wyczesany (2017) further found that stimuli with negative features elicited stronger attentional processing. These findings indirectly support the view that informational conflict is subject to sustained unconscious processing and suggest that the content of the information may influence the degree of such processing. In addition, perceptual conflict may not only produce transient interference within the current trial, but may also persist and modulate processing in subsequent trials, thereby exhibiting a form of cross-trial regulation (Zhao and Wang, 2013). This suggests that perceptual conflict may have a sustained regulatory role independent of response execution and goal-directed processing, and that its effects may extend into higher-level cognitive control systems and influence subsequent information-processing strategies.

The Cascade-of-Control model proposed by Banich (2019) further enriches this perspective. According to this model, conflict in the Stroop task is not confined to the response stage. Instead, it may arise successively across multiple stages of information processing, from stimulus recognition and attentional selection to conflict detection, response mapping, and execution, with each stage involving a dynamic allocation of control resources. More importantly, the model proposes that the degree of control deployed at an early stage influences the amount of control required at later stages, indicating a cascading and forward-linked mechanism of resource allocation. More specifically, this sustained influence may first be registered by the monitoring system as a processing state that requires additional regulation. Conflict monitoring theory holds that the cognitive system does not initiate regulation only after control has failed. Rather, once informational conflict is detected, it generates a signal that enhanced control is required (Botvinick et al., 2001). Neuroimaging evidence from the Stroop task likewise shows that conflict-related activity in the anterior cingulate cortex can predict subsequent increases in prefrontal activity and later behavioral adjustment, suggesting that conflict information detected at an early stage can further shape subsequent control configuration (Kerns et al., 2004). In this sense, the perceptual conflict effect preserved in the Task-Irrelevant Paradigm is better understood as a process that begins with early inconsistency detection and may extend into subsequent stages of monitoring and regulation.

When considered together with findings from the Task-Irrelevant Paradigm, this perspective further suggests that even if perceptual conflict does not directly affect response selection, the early interference and cognitive regulation it induces may continue to shape subsequent attentional allocation and processing efficiency. Neurologically, this is reflected in later ERP components such as the N450 and LPC. This view is closely aligned with the multi-stage control framework emphasized in Banich’s model and provides a useful theoretical basis for understanding the sustained influence of perceptual conflict within the Task-Irrelevant Paradigm.

#### 2.2.3 Encoding mechanisms based on processing depth

Given the characteristics of informational conflict and task conflict in the Stroop paradigm, it is generally assumed that Stroop interference arises from competition between response channels caused by differences in the processing speed of stimulus information (Doehrman et al., 1978; Posner and Snyder, 2004). This view is rooted in theories of automaticity in human information processing. Early work by Cattell (1886) is often regarded as foundational, and later theoretical developments by LaBerge and Samuels (1974), Shiffrin and Schneider (1977), and Logan (1978) further elaborated this account. These studies proposed that word information can directly initiate a response, whereas color information must first be transformed from a visual representation into a phonological one. As a result, word information reaches the response stage faster than color information, thereby producing interference and response delays (Doehrman et al., 1978).

However, MacLeod (1991, 1992) pointed out that although this account can explain most key findings related to the Stroop effect, it does not hold up in the most direct experimental tests, namely those that manipulate processing speed through SOA (stimulus onset asynchrony) or practice. MacLeod further criticized automation theory as providing only an incomplete explanation of the Stroop effect, especially with regard to the relationship between strong automaticity, defined as processing unaffected by attentional strategies, and the Stroop effect. He proposed that the notion of “partial automaticity” may be more appropriate for describing the Stroop effect. Therefore, the explanation based on differences in stimulus encoding speed does not yet have sufficient empirical support.

The theoretical ambiguity surrounding encoding mechanisms stems fundamentally from the inadequate separation of the two types of conflict in typical conflict interference paradigms. From the perspective of the Task-Irrelevant Paradigm, this issue can be clarified to some extent. The influence of differences in processing speed appears to be expressed mainly in competition between response tendencies and in conflict resolution (Doehrman et al., 1978). By contrast, perceptual conflict in the Task-Irrelevant Paradigm is shaped primarily by the level of stimulus encoding during holistic conflict processing (Oren et al., 2023).

Two ERP studies using the Task-Irrelevant Paradigm provide empirical support for this distinction, despite yielding different behavioral results (Li et al., 2013; Yuanqing et al., 2024). In Li et al. (2013), a Stroop-like task-irrelevant design was used in which participants judged the rotational state of the stimuli; this is referred to here as the rotation task. By contrast, Yuanqing et al. (2024) used a Navon-like task-irrelevant design in which participants judged the orientation of local elements within compound stimuli; this is referred to here as the direction task. Both studies examined the effects of perceptual conflict on the same ERP components while also analyzing behavioral performance. Both found that perceptual conflict increased the N450 component and the Late Positive Complex (LPC). The key difference lay in the behavioral outcomes: the rotation task showed no significant conflict effect, whereas the direction task did. In other words, perceptual conflict in the rotation task did not produce a significant behavioral interference effect. A comparison of the experimental conditions in these two studies suggests three possible sources for this discrepancy.

1. In the rotation task, judging whether a word is rotated can be accomplished by attending to the orientation or overall shape of the text, without requiring detailed word reading. Accordingly, this task likely involves a relatively low level of semantic activation. In contrast, the direction task requires participants to determine whether the word is upright, which demands more precise word recognition. This process is more likely to engage deeper semantic processing and, in turn, elicit awareness of informational conflict.

2. In the rotation task, the experimental stimuli consist of color and word meaning and mainly involve the perception of physical attributes. This places a relatively low cognitive load on participants and remains largely within the perceptual domain. This suggests that although perceptual conflict is present, it may not extend into deeper cognitive or social processing stages and therefore may have only a limited effect on task performance. In contrast, the stimuli used in the direction task, such as combinations of geometric shapes and social roles, carry richer sociocultural meaning. These shapes and roles may be more deeply rooted in participants’ cognitive systems, functioning not merely as objects of visual recognition but also as symbols associated with personal experience and cultural context. Consequently, this deeper level of information processing may increase cognitive load and affect task execution.

3. In the rotation task, all stimuli, including both upright and rotated items, were included in the data analysis. This may have introduced additional noise related to rotation status, because rotated words can increase reading difficulty and potentially mask the effects of deeper perceptual conflict. In contrast, the direction task analyzed only upright stimuli, thereby reducing the additional cognitive load that may be induced by atypical word configurations, such as inverted text (Kao et al., 2010). The visual and semantic processing of upright words is generally more direct and clearer, making this selection more effective for revealing the effect of perceptual conflict on task performance in the Stroop effect.

According to levels-of-processing theory (Chwilla et al., 1995; Craik and Lockhart, 1972), perception is not a passive reception of environmental stimuli but is shaped by the depth of information processing. Accordingly, the cognitive resources required by a task and the depth of information processing can substantially affect the final processing outcome (Daniel and Kapoula, 2019; Wu and You, 2017; Craik and Tulving, 1975). Compared with the direction task, the rotation task is less demanding, and its stimuli mainly involve the perception of physical attributes, resulting in a relatively low cognitive load. By contrast, the direction task requires more attentional resources, which may facilitate higher-level information processing of the stimuli. As a result, incongruent stimuli may not only increase cognitive load but also activate error-detection processes (Bekçi and Karakaş, 2009). Similarly, Burca et al. (2021) reported clear evidence that perceptual conflict contributes to the overall Stroop effect. Thus, the cognitive resource load induced by perceptual conflict may produce a Stroop effect even in the absence of response conflict.

In addition, Wolska (2021) adopted a Navon-like Task-Irrelevant Paradigm and analyzed the conditions under which an irrelevant task produces interference. The results showed that interference from the irrelevant task does occur and is independent of letter identity and response speed. Other studies using the Task-Irrelevant Paradigm likewise confirmed that when a task requires deep processing of a conceptually unrelated dimension, such as animacy judgment, interference from conflict-related information may still occur even if that information is irrelevant to the task (Hatukai and Algom, 2017; Oren et al., 2023).

Recent studies have further shown that the depth of stimulus encoding significantly affects the interference effect of perceptual conflict. For example, Schreiter et al. (2019), integrating EEG data with pupil-diameter measures, found that the emotional Stroop effect became stronger as the processing depth of emotional stimuli increased. This suggests that processing depth plays an important role in the interference effect of emotional information. Pan et al. (2019) further showed that actively maintaining color words in working memory can produce interference effects similar to those found in the typical Stroop effect, highlighting the importance of internal maintenance mechanisms for processing depth. Similarly, Sharma et al. (2019), examining the neural electrical activity of musicians performing an auditory Stroop task, found that professional background and skill level, such as absolute pitch, enhanced the processing of conflicting information and thus influenced Stroop performance. Chen and Zou (2021) also used second-language words as Stroop stimuli and found that participants with higher second-language proficiency took longer to name colors and showed lower accuracy, indicating that the linguistic encoding level of stimuli significantly affects Stroop task performance.

In summary, perceptual conflict appears to affect task performance substantially only when information processing reaches a certain depth. Deeper information processing not only increases cognitive load but may also amplify the influence of perceptual conflict. Through refined experimental design and condition adjustment, researchers may be able to manipulate the strength of perceptual conflict more effectively within the Task-Irrelevant Paradigm. This, in turn, may allow a more precise examination of the specific role of perceptual conflict in cognitive processing and deepen our understanding of its effect on cognitive task performance.

#### 2.2.4 Processing mechanism based on selective attention and modular perspective

In the Task-Irrelevant Paradigm, once the conflict dimension is dissociated from the current task goal and response conflict is largely excluded, it becomes possible to examine the composition of the remaining effects more closely. It is therefore necessary to distinguish more carefully between two questions. First, do these effects arise because the internal conflict relation within the stimulus is still extracted automatically even when it falls outside the current task goal? Second, do these early conflicts, and under what conditions, further develop into observable behavioral costs? From the perspective of cognitive control, Oberauer (2019) argued that attention can be understood both as a selection mechanism and as a limited resource. On this basis, the following discussion focuses on two issues: why conflict information in Task-Irrelevant Paradigm can still enter the processing system, and why such conflict sometimes remains at an early or implicit stage but at other times becomes visible in reaction time or accuracy differences.

From the perspective of selective attention, task-irrelevance does not mean that processing cannot occur. Posner’s (1980) work on orienting attention showed that attention can be directed preferentially to a particular location or object even without eye movements, thereby increasing the likelihood that information within that range will be detected and further analyzed. This implies that as long as the conflict dimension remains within the attended stimulus structure, it will not automatically be excluded from perceptual selection simply because it is not part of the current task goal. Perceptual load theory further suggests that whether distractors are processed is determined not only by their relevance to the task, but also by the type and level of the current processing load: high perceptual load typically reduces distractor interference, whereas increased working-memory load or dual-task coordination demands may increase vulnerability to interference (Kane and Engle, 2003; Lavie et al., 2004; Lavie, 2005). Consistent with this view, Forster and Lavie (2008) found that completely irrelevant distractors could still affect task performance, although this effect was significantly reduced under high perceptual load. Likewise, an fMRI study by Banich et al. (2001) showed that the processing of task-irrelevant attributes is modulated by attentional selection mechanisms. Accordingly, after response conflict has been removed, the residual effects observed in Task-Irrelevant Paradigm can at least partly be explained by the continued entry of conflict information into early processing stages.

On this basis, a modular perspective provides a further explanation. If different types of information processing correspond to relatively content-specific processing units, then a given dimension may still be extracted automatically within its corresponding processing unit even when it is not part of the current task goal. However, this does not mean that such processing modules are fully independent of one another (Wickens, 2008; Oberauer, 2019). Multiple resource theory proposes that different processing operations may be partly separate and partly overlapping in modality, stage, coding format, and response channel, and that interference often emerges when multiple processes compete for the same resources (Wickens, 2008). Accordingly, the effects observed in Task-Irrelevant Paradigm do not support the view that different forms of information processing are completely sealed off from one another. A more plausible interpretation is that the congruent or incongruent relation within a stimulus can first be extracted automatically within content-relevant processing modules, while these modules still partially overlap at higher levels of attention and control. Previous research has shown that the congruity/incongruity relation within Stroop stimuli affects not only the classic task dimensions but also judgments on an “additional dimension,” indicating that relational information within the stimulus can influence processing beyond the current task dimension (Hatukai and Algom, 2017). Oren et al. (2023) further used a judgment task defined at a different conceptual level from the conflict dimension and found that incongruent stimuli still produced longer reaction times even when the conflict was irrelevant to the current task; moreover, this behavioral congruency effect was associated with activity in the bilateral intraparietal sulcus. At the same time, Algom and Chajut (2019) argued that Stroop-like effects should not be reduced too simply to late control processes, and that greater attention should be paid to the role of input-stage processing, dimensional relations, and perceptual selection. Taken together, these findings suggest that the effects produced in Task-Irrelevant Paradigm are initially closer to a form of content-specific information extraction than to mere global slowing or undifferentiated resource depletion.

However, the emergence of an early conflict signal does not mean that it will necessarily become behavioral interference to the same degree. Capacity theory treats attention as a limited and shared processing resource (Kahneman, 1973), with the key point being that attentional resources are not unlimited but must be allocated across tasks (Bruya and Tang, 2018). When this view is combined with the modular perspective outlined above, it becomes possible to explain further why conflict information may first be triggered within relatively content-specific processing modules, but whether it is ultimately expressed in reaction time or accuracy depends on whether these early processes continue to consume attentional and control resources that partially overlap with those required by the current task. Research on the semantic Stroop effect also supports this interpretation. Sulpizio et al. (2024) found that semantic Stroop interference depends to some extent on available executive resources and that its distribution changes under an additional n-back load, suggesting that whether early conflict becomes behaviorally manifest is regulated by the availability of proactive control resources. Thus, the effects produced in Task-Irrelevant Paradigm appear to have a “content-triggered, resource-gated” structure: they begin with the content-specific extraction of relational information within the stimulus, whereas the degree to which they become behaviorally visible is regulated by the state of shared attentional and control resources. What resource limitation mainly determines is not whether conflict is triggered, but whether that conflict can further develop into a behavioral effect.

In addition, conflict being detected, conflict entering conscious representation, and conflict further triggering control adjustment are not three fully overlapping processes. Previous research has shown that even when stimulus visibility is restricted, the experience of conflict can still alter behavior on subsequent trials, suggesting that the monitoring system’s registration of inconsistent information does not necessarily require full explicit awareness (van Gaal et al., 2010). By contrast, research on error awareness shows that conscious awareness of an error or conflict state is usually accompanied by stronger autonomic arousal and a clearer tendency toward subsequent adjustment, giving it a functional meaning closer to the formation of a reportable higher-level representation of the current processing state (Ullsperger et al., 2010).

In summary, in Task-Irrelevant Paradigm, after response conflict has been removed, the remaining effects can first be understood as reflecting the automatic extraction of internal conflict relations within the stimulus even when those relations fall outside the current task goal. Whether this early conflict is further expressed as stable behavioral interference depends on the partial overlap among modules at higher levels of attention and control resources. This is also why the Task-Irrelevant Paradigm is more useful than traditional conflict-stimulus paradigms for disentangling the internal processing mechanisms of conflict effects, rather than merely demonstrating that some form of interference still exists.

### 2.3 Application potential and limitations of the task-irrelevant paradigm

The core contribution of the Task-Irrelevant Paradigm lies in its ability to provide a relatively pure method for measuring perceptual conflict effects. Compared with other conflict interference tasks, this approach has been used more extensively in Stroop tasks and offers a new way to disentangle the overlapping effects of multiple types of conflict in conventional paradigms.

However, despite its strong explanatory value and discriminative capacity, research using this paradigm is still relatively limited. Concerns remain regarding the stability and reproducibility of experimental findings, and its operational standards and evaluation frameworks have not yet been widely validated. Even so, given its advantages in terms of core characteristics, the Task-Irrelevant Paradigm continues to show considerable potential for future application.

#### 2.3.1 Application potential

##### 2.3.1.1 Automatic processing and resistance to strategic interference

One of the key advantages of the Task-Irrelevant Paradigm is that it engages the perceptual system’s automatic processing mechanisms. Compared with typical paradigms, this form of implicit conflict processing is less likely to be shaped by deliberate control strategies or task training. According to dual-process theory, controlled processing typically depends on the maintenance and regulation of task goals. By placing the conflict dimension outside the current task goal, the Task-Irrelevant Paradigm reduces the coupling between goal-directed processing and the conflict effect under investigation, making it better suited to capturing the automatic extraction of conflict information and its downstream effects.

Studies have shown that in typical Stroop tasks, the close coupling between task goals and interference information—for example, color naming in the presence of word meaning—often leads participants to develop control strategies across repeated trials, such as actively suppressing word processing or increasing attentional focus on color. These strategies can, in turn, modulate the emergence of task conflict (Kalanthroff et al., 2013, 2018). In contrast, the Task-Irrelevant Paradigm places conflict-related information outside the scope of the task goal, giving participants little reason or opportunity to exert conscious control. Under these conditions, the activation of conflict information relies primarily on automatic perceptual processing with minimal conscious awareness, and thus may better reflect individuals’ natural response patterns in the absence of strategic intervention.

More importantly, because the Task-Irrelevant Paradigm removes task-driven attention to conflict-related information, the processing it elicits may more closely approximate situations in everyday life in which individuals encounter multidimensional informational interference. Compared with typical paradigms, it may therefore offer greater ecological validity for examining how the cognitive system detects and responds to environmental conflict under relatively natural conditions. In this sense, a perception-based and relatively automatic conflict detection process may provide a useful experimental model for understanding how implicit interference affects the cognitive system in real-world contexts.

##### 2.3.1.2 Implicit associations between object properties

In everyday cognition, individuals often develop implicit structural associations across perceptual dimensions through long-term experience, such as associations between color and shape, category and function, or spatial location and meaning (Zelazny et al., 2023). These associations are shaped not by explicit task rules, but by extended life experience and semantic schemas. They give rise to a form of “perceptual expectation” or “coherence intuition” about object structure (Schloss, 2024; Mukherjee et al., 2021). Within a predictive-processing framework, the brain continuously generates predictions about sensory input and updates them to minimize prediction error (Clark, 2015). Consistent with this account, Cheng et al. (2021) reported that ERP markers of perceptual conflict are driven primarily by long-term stimulus–stimulus associations rather than by transient associations formed during short-term experimental tasks.

By decoupling task goals from the conflict dimension, the Task-Irrelevant Paradigm can more effectively reveal whether participants automatically extract consistency or inconsistency information embedded in stimuli when the conflicting dimension is not task-relevant, thereby eliciting perceptual conflict. When such coherence is disrupted, conflict may arise automatically even if the conflicting attribute is not directly processed. This makes it easier to identify which stimulus properties are implicitly linked at the level of object structure. For this reason, the paradigm is particularly well suited to examining whether implicit associations exist between object features.

##### 2.3.1.3 Metaphorical processing in social cognition

In social cognition, people’s understanding of others, contexts, and symbols often relies on cross-dimensional metaphorical associations, such as “a warm person” or “a superior leader.” These associations are widespread in language and also show strong psychological automaticity at the perceptual level (IJzerman et al., 2012). For example, the association between red and danger appears not only in linguistic imagery but also in emotional evaluation and attentional allocation in experimental tasks (Fetterman et al., 2012). Likewise, structural associations among social symbols, such as roundness–gentleness and face–gender, reflect implicit encodings shaped by long-term learning of social meaning (Blazhenkova and Kumar, 2018). These associations are often culturally shared and relatively stable across contexts, giving rise to fairly consistent encoding tendencies within a given group.

For this reason, such cross-dimensional metaphorical associations are particularly suitable for investigation within the Task-Irrelevant Paradigm. By separating the task goal from the metaphorical dimension, this paradigm reduces the need for participants to deliberately retrieve relevant knowledge or make associative judgments, and thus is better able to capture the automatic activation of social metaphors rather than the products of strategic reasoning.

Furthermore, typical conflict interference paradigms often heighten participants’ explicit attention to possible relationships between stimuli, thereby introducing higher-order influences such as subjective reasoning, social expectations, or task strategies. In contrast, under the Task-Irrelevant Paradigm, the social-symbolic associations embedded in the stimuli fall outside the focus of task-directed attention. If conflict effects are still observed under these conditions, this provides stronger grounds to infer that the interference reflects relatively automatic perceptual detection of symbolic conflict, rather than task-induced linguistic strategies or conceptual reasoning (e.g., Yuanqing et al., 2024).

#### 2.3.2 Limitations

##### 2.3.2.1 Instability of perceptual conflict effects; limited empirical support

At present, relatively few studies have directly examined the Task-Irrelevant Paradigm and its applications. Moreover, existing empirical findings show that behavioral effects are not consistent across experiments. Under some conditions, no significant behavioral effects have been observed (Li et al., 2013; Wolska, 2021), whereas in other cases, incongruity effects have even been reported (Hatukai and Algom, 2017).

Several factors may contribute to this inconsistency.

First, the task-irrelevant manipulation may not be well matched to the presentation format of the conflict stimulus. As a result, conflict-related information may fail to enter deeper levels of encoding and instead remain at a relatively superficial level of processing, thereby failing to produce detectable interference.

Second, effects in the Task-Irrelevant Paradigm are likely to be smaller than those observed in typical paradigms. This is because the paradigm removes stronger sources of conflict while at the same time attempting to detect relatively automatic perceptual processing.

Third, insufficient precision in the setting of experimental parameters may cause these already weak effects to be masked by noise.

##### 2.3.2.2 Lack of a standardized framework

The Task-Irrelevant Paradigm has not yet developed a standardized methodological framework. At present, it still largely depends on experimental stimuli derived from foundational paradigms such as the Stroop, Flanker, and Navon tasks, with corresponding modifications to task operations. In addition, apart from manipulation of the task dimension, no empirical marker has been established that is specific to the Task-Irrelevant Paradigm. In the absence of a consensus framework with clear criteria for specificity and sensitivity, task-irrelevant conditions can be constructed in highly diverse ways, which in turn reduces comparability across studies. From another perspective, however, this flexibility may also create room for further development.

Additionally, although the paradigm appears to be broadly applicable to conflict interference research and has so far been used mainly in Stroop-like and Navon-like forms, its overall applicability still requires further empirical validation. In light of these limitations, whether researchers are constructing new task-irrelevant conditions or extending existing paradigms to other research domains, careful and precise implementation remains essential.

## 3 Research prospect

Research on perceptual conflict within the Task-Irrelevant Paradigm remains at an early stage. Only a limited number of studies have adopted this paradigm, and clarifying the design-related factors that shape its effects will be important for both theoretical development and methodological refinement. At the same time, multimodal integration can enrich the evidence base beyond that available in traditional psychological experiments. This offers clear advantages for building a stable and standardized operational framework and for examining conflict effects with greater precision. Future research on the Task-Irrelevant Paradigm and perceptual conflict effects may therefore proceed along the following lines.

### 3.1 Factors affecting the task-irrelevant paradigm

The Task-Irrelevant Paradigm plays a key role in isolating response conflict and thus allows a more focused examination of perceptual conflict. It also provides a useful framework for analyzing the mechanisms underlying conflict interference effects. However, perceptual conflict effects observed in this paradigm have been relatively unstable (Li et al., 2013; Wolska, 2021). It remains unclear how perceptual conflict interferes with either task-relevant or task-irrelevant processing, and behavioral findings vary considerably across studies. For example, Wolska (2021) reported that both stimulus parameter settings and the form of the irrelevant task significantly affected the observed behavioral outcomes. Two factors may contribute to the attenuation of these effects. First, unlike typical paradigms, the Task-Irrelevant Paradigm includes only perceptual conflict and does not directly elicit response conflict. Consequently, it produces little or no interference at the response selection stage. Second, in typical paradigms, overlap between the task goal and the conflict dimension increases the activation of perceptual conflict, whereas no such overlap is present in the Task-Irrelevant Paradigm. Prior findings suggest that when response conflict is removed from the Stroop effect, the observed effect may be reduced by half or even more (Burca et al., 2021; Li et al., 2013).

Beyond these two sources of attenuation, perceptual conflict in the Task-Irrelevant Paradigm may also be shaped by several additional factors. First, semantic associations between dimensions (e.g., words and colors) can influence attentional allocation (Dishon-Berkovits and Algom, 2000). Even in a task-irrelevant context, such associations may lead participants to process conflicting information unintentionally, and stronger associations may produce stronger activation of perceptual conflict. Second, stimulus salience and task complexity can also affect conflict strength (Donohue et al., 2013; Yamagishi and Furukawa, 2020). When stimulus features are more visually salient, participants may find it harder to ignore prominent task-irrelevant information, thereby increasing perceptual conflict. By contrast, when task difficulty is higher, greater cognitive control is required, which may reduce the impact of conflict on performance (Folk et al., 1992). The dual-process framework distinguishes this process into proactive control and reactive control: the former involves the sustained maintenance of stimulus information, whereas the latter refers to the transient recruitment of control after conflict emerges (Braver, 2012). In Stroop research, concurrent working-memory load weakens proactive control and leads to greater interference and more pronounced task conflict, suggesting that the way cognitive resources are allocated can substantially alter the likelihood that conflict progresses from early detection to overt behavioral expression (Kalanthroff et al., 2015). In the Task-Irrelevant Paradigm, such working-memory load may reduce the detectability of perceptual conflict.

Based on the foregoing analysis of the Task-Irrelevant Paradigm, we suggest that researchers should pay particular attention to two issues when applying this paradigm.

First, the depth of stimulus encoding should be carefully regulated through both task demands and the characteristics of the experimental materials. On the one hand, task demands exert a top-down influence on the activation of target stimuli. For example, in the rotation task used by Li et al. (2013), participants made judgments solely on the basis of word orientation and did not need to fully read the word itself. As a result, semantic activation was limited, reducing the potential for perceptual conflict. By contrast, the orientation task used by Yuanqing et al. (2024) required participants to judge whether a character was upright, which ensured full visual processing of the word and increased the likelihood of observing conflict effects at the behavioral level. Oren et al. (2023) further suggested that the task should require deeper semantic analysis or conceptual judgment of the stimulus rather than mere processing of surface features. Accordingly, when using the Task-Irrelevant Paradigm, researchers should design tasks that engage key aspects of the target stimulus sufficiently to elicit more salient perceptual conflict.

On the other hand, the cognitive complexity and salience of the experimental materials may exert bottom-up effects on stimulus encoding (Wolska, 2021). For example, tasks focused on low-level perceptual attributes tend to produce weaker conflict effects (Hatukai and Algom, 2017; Li et al., 2013; Wolska, 2021; Zhao and Wang, 2013), whereas tasks involving semantic processing or stimuli with richer socio-cultural meaning are more likely to yield robust conflict effects. Researchers designing Task-Irrelevant Paradigm experiments should therefore consider using materials that contain richer informational content and are more likely to engage deeper cognitive processing. To address dimensional associations, asymmetric task designs may also be useful. For instance, non-standard color words, abstract shapes, or words associated with color rather than conventional color terms could be used (Entel and Tzelgov, 2018; Hershman et al., 2021), thereby reducing automatic responses driven by strong word-color associations. To control stimulus salience and task complexity, visual attributes such as font size and color should be standardized across stimuli to minimize their influence on task performance (Theeuwes, 1992; Wolska, 2021). In addition, incorporating multi-step decision processes or time pressure may lead participants to rely more on intuitive than deliberative processing (Entel et al., 2015). Such designs may strengthen automatic stimulus processing and thereby facilitate stronger activation of perceptual conflict during cognitive processing.

Second, experimental data processing should be refined, particularly with respect to the number of trials and the exclusion of outliers. Because the Task-Irrelevant Paradigm tends to produce smaller effect sizes than typical paradigms, the number of trials should be increased appropriately. This can help ensure that even subtle conflict effects are detectable at the statistical level. A larger number of trials can improve statistical power, enhance the reliability of the results, and increase the precision of effect-size estimates. These design choices are important for ensuring that the findings rest on an adequate empirical basis. In addition, rigorous criteria should be used during data analysis to exclude abnormal data. For example, in the rotation task, Li et al. (2013) included all stimuli, including both upright and rotated words, in the analysis. Although this strategy increased the number of trials, it also introduced additional noise, because rotated words may be more difficult to read and may therefore obscure or dilute deeper perceptual conflict effects. By contrast, in the orientation task, Yuanqing et al. (2024) analyzed only upright words, thereby avoiding the additional cognitive load induced by inverted text (Kao et al., 2010). This strategy allowed a clearer distinction between normal and atypical reading conditions, so that the resulting data more directly reflected the effects of perceptual conflict. Accordingly, within the Task-Irrelevant Paradigm, more precise and adaptive data-analysis strategies are recommended to better capture how cognitive conflict is processed.

### 3.2 Extending the task-irrelevant paradigm across paradigms: structural conditions and theoretical implications

From the overall structure of classic conflict interference tasks, many paradigms contain both task-relevant and task-irrelevant information. However, the interference elicited by these sources of information does not always arise at the same processing level. According to dimensional overlap theory, the relevant and irrelevant dimensions in a task may form compatibility relations at the stimulus–stimulus level, or they may primarily affect response selection and execution at the stimulus–response level (Kornblum et al., 1990). Accordingly, although Stroop, Flanker, Simon, and Go/No-Go tasks can all be classified broadly as conflict tasks, they differ in the source of conflict, the stage of processing involved, and the theoretical issues they address (Kleinsorge, 2021). Comparing different types of conflict tasks and their variants can therefore reveal both common and distinct features of cognitive processing and deepen our understanding of cognitive control in conflict resolution. A central question for extending the Task-Irrelevant Paradigm is whether a given paradigm structurally allows the current task goal to be shifted away from the original conflict dimension while preserving the original conflict relation, so that the residual effect remains interpretable.

From this perspective, whether a paradigm can be combined with the Task-Irrelevant Paradigm depends on at least several structural conditions. First, the paradigm itself must contain a conflict relation that can be preserved rather than eliminated entirely by task switching. Second, the stimulus must include an independent dimension that can carry the task requirement, and this dimension must not belong to the same conceptual dimension as the original conflict dimension. Third, after the task is shifted to this independent dimension, the original paradigm should still yield interpretable behavioral or neural indices, rather than simply turning into a basic discrimination task unrelated to conflict. Finally, the residual effect retained after such a modification should still be open to further localization as reflecting perceptual competition, response coding, or inhibitory control. By these standards, paradigms differ in both how readily they can be combined with the Task-Irrelevant Paradigm and what theoretical value such a combination may have.

Beyond the Stroop-like and Navon-like task-irrelevant designs discussed in this paper, the Flanker paradigm appears to be structurally well suited to this approach. It naturally contains a stimulus structure in which target and distractor elements coexist, and the target-flanker congruent/incongruent relation can be preserved after a task shift. This makes it relatively easy to introduce an additional dimension to carry the current task. For example, participants could be asked to judge the color, brightness, or outer-frame shape of the target item while the original target-flanker congruent/incongruent relation is retained as a task-irrelevant structure. In this way, the original conflict is not removed but shifted from the response-defining dimension to the background structure of the stimulus. Existing research shows that even when color serves as a task-irrelevant dimension, it can still consume attentional resources and modulate both behavioral performance and ERP indices in the Flanker task (Chen et al., 2024). In addition, ERP studies using a 2-to-1 mapping design indicate that Flanker interference is not a unitary component but includes both conflict and distraction, involving early attentional processing, later conflict evaluation, and response inhibition (Zhou et al., 2019). Structurally, then, the Flanker paradigm is not only easy to combine with a task-irrelevant design, but is also particularly useful for asking whether, once the original congruent/incongruent relation no longer directly determines the response, the remaining interference mainly reflects perceptual competition, attentional allocation, or continued transmission to the response stage.

Compared with the Flanker task, paradigms such as the Simon task, spatial Stroop, and orthogonal SRC can also be adapted, but their functional role is different. What these tasks share is that task-irrelevant spatial location automatically activates response codes and thereby affects performance in visual choice-reaction tasks (Lu and Proctor, 1995). Structurally, these paradigms can be modified by shifting the current judgment to a nonspatial dimension while preserving the spatial compatible/incompatible relation. Even so, the irrelevant spatial dimension often continues to exert a strong pull on response codes. In other words, the residual effect preserved after such adaptation is still likely to reflect automatic activation at the response-selection level, rather than a relatively pure form of perceptual conflict. The study by Scerrati et al. (2017) supports this distinction. After comparing Stroop-like and Simon effects, they found that the two could show additive behavioral effects and different temporal distributions, suggesting that Stroop-like and Simon effects are more likely to arise at different processing stages. These paradigms may therefore be better suited to investigating response coding in conflict processing, and to examining how the structural link between an irrelevant dimension and response codes continues to shape response selection.

The Go/No-Go paradigm represents another, more borderline case. From a design perspective, it is not impossible to combine it with the Task-Irrelevant Paradigm, but the conditions for doing so are clearly more restrictive than in the Flanker paradigm. The basic reason is that the core of Go/No-Go is not the congruent or incongruent relation between stimuli itself, but the interplay between a prepotent response and response inhibition. Accordingly, introducing a task-irrelevant design into Go/No-Go usually requires an additional dimension to carry the response rule. For example, color, a frame, or another neutral feature could determine the go/no-go response, while emotional content, semantic relations, or other potentially conflicting information is retained as the task-irrelevant dimension. Under such a design, task-irrelevant information may still influence processing, but it is difficult to strip away the contribution of response control as fully as in a Stroop-like design. Existing research shows that introducing task-unrelated emotional information into a Go/No-Go task can indeed alter behavioral performance as well as the no-go N2/P3 components (Chen et al., 2022). At the same time, Donkers and Van Boxtel (2004) showed that no-go N2 is more likely to reflect conflict monitoring than mere response inhibition. Taken together, these findings suggest that once Go/No-Go is combined with a task-irrelevant design, it is better suited to examining whether early conflict or task-irrelevant information is transmitted downstream to modulate inhibitory control, rather than serving directly as a tool for extracting relatively pure perceptual conflict.

Overall, the relation between a given paradigm and a task-irrelevant design depends on whether its task structure allows the original incongruent relation to be preserved while the mapping between the task goal and the conflict dimension is reassigned. For this reason, extending the Task-Irrelevant Paradigm can be understood as a comparative framework. It allows researchers to examine, across different task structures, the perceptual integration of conflict information and the inhibitory control involved in response coding.

### 3.3 Multimodal integration research

Recent research on cognitive conflict has increasingly integrated multimodal techniques, including functional magnetic resonance imaging (fMRI), event-related potentials (ERP), and eye-tracking, to investigate the physiological basis of conflict interference effects. Against this background, combining the Task-Irrelevant Paradigm with Stroop-like tasks may provide a useful approach. In such designs, participants respond to attributes unrelated to Stroop conflict, such as the size or shape of letters, rather than to word meaning or color. This approach can help dissociate perceptual conflict from response conflict and thus support more precise identification of the physiological processes underlying perceptual conflict in the Stroop effect.

In addition, in Stroop research, novelty effects elicited by incongruent stimuli often complicate the interpretation of genuine conflict effects. Although the Task-Irrelevant Paradigm can reduce response delays caused by conflict-related attributes, novelty-related interference may still remain. Standardized experimental design and pre-experiment training are often used to control such interference, but they may not fully eliminate it. Multimodal methods can help address this problem by providing converging physiological evidence from different levels of analysis. This can strengthen the interpretation of experimental results and clarify how novelty effects interact with conflict processing. More broadly, such work may contribute to both the theoretical development and the methodological refinement of the Task-Irrelevant Paradigm, while improving our understanding of perceptual conflict in complex cognitive tasks.

ERP offers high temporal resolution, allowing millisecond-level tracking of processing from early perceptual analysis—for example, the detection of mismatched color-word information—to later response selection, such as the suppression of conflicting information. It can therefore reveal neural components associated with different stages of cognitive processing. For example, components such as P2, N450, and LPC (Late Positive Complex) have been linked to different phases of conflict monitoring and decision-making, helping to identify when and how conflict arises (Di Russo and Bianco, 2023; Galashan et al., 2019; Heidlmayr et al., 2020; Rey-Mermet et al., 2019). Novel stimuli also often elicit a larger P300, suggesting enhanced processing of unexpected events (Folstein and Van Petten, 2008).

fMRI offers high spatial resolution and can help identify the brain regions engaged during Stroop-task processing. This makes it possible to distinguish neural activity related to perceptual conflict from that related to response conflict (Donohue et al., 2016). Under the Task-Irrelevant Paradigm, fMRI may reveal activation patterns that differ from those found in typical paradigms (Hatukai and Algom, 2017). For example, reducing response conflict through task irrelevance may decrease activity in regions involved in conflict resolution while increasing activity in regions associated with the alternative task demands. Novel stimuli are typically associated with greater activation in the nucleus accumbens, frontal cortex, and parietal regions, which are linked to attentional reallocation and reward systems (Krebs et al., 2011). By contrast, conflict effects are often associated with the anterior cingulate cortex (ACC) and prefrontal cortex (PFC), which play central roles in monitoring and resolving cognitive conflict (Kerns et al., 2004). Together, these findings and hypotheses help clarify the cognitive functions and neural mechanisms involved in interference tasks and provide empirical support for distinguishing different forms of conflict and refining models of cognitive control.

Finally, eye-tracking can provide useful information about how participants allocate visual attention and resolve conflict in the Task-Irrelevant Paradigm. This can further clarify the contribution of attention to both perceptual and response conflict (Gardumi et al., 2016). For example, perceptual conflict is often associated with longer fixation durations (Egner and Hirsch, 2005), whereas response conflict tends to involve more frequent saccades, especially when response inhibition or the overriding of automatic responses is required (Schulte et al., 2009). Novel stimuli often produce higher regression rates and stronger initial fixation responses (Krebs et al., 2013).

Computational neural modeling can also be used to parameterize factors that influence conflict effects. For example, by incorporating the Drift Diffusion Model (DDM), researchers can quantify cognitive load more precisely during the processing of congruent and incongruent stimuli in the Task-Irrelevant Paradigm through parameters such as drift rate and decision boundary. Studies have shown that a lower drift rate may reflect slower perceptual processing of incongruent stimuli, whereas a higher decision boundary may indicate that more information is required for an accurate decision (Shinn et al., 2020). Other studies have reported that DDM parameters may correlate with ERP components such as the N400, suggesting a link between model-based estimates of semantic conflict and neural markers of semantic processing (Freund et al., 2021; Swerdloff and Hargrove, 2023). These findings suggest that combining DDM outputs with ERP or fMRI data may provide deeper insight into changes in brain activity during conflict processing.

Despite its theoretical promise, this multimodal approach still faces several practical limitations. Current multimodal research remains focused mainly on the mechanisms of basic color-word Stroop tasks and is less often extended to more complex or more ecologically valid designs. Several studies have recommended expanding the use of fMRI, ERP, and eye-tracking to a wider range of task conditions (Hershman et al., 2022; Yousef Pour et al., 2020; Rizzo et al., 2022; Simeonova et al., 2022; Wang et al., 2021). These developments may also benefit research using the Task-Irrelevant Paradigm by extending multimodal methods to a wider range of task conditions.

Moreover, experimental design remains a major challenge in multimodal integration research. To obtain reliable data, experiments must be designed so that multiple technologies can be synchronized and can effectively capture participants’ behavior and neural activity during Task-Irrelevant Paradigm tasks. This requires careful coordination between the technical characteristics of each modality and the demands of the task to ensure compatibility across measures. Even so, multimodal approaches remain valuable for research on the Task-Irrelevant Paradigm. By integrating evidence from different techniques, researchers may better characterize the cognitive and neural processes involved in conflict resolution. This, in turn, may support theoretical advances in cognitive science and provide an empirical basis for cognitive assessment and training grounded in conflict interference effects.

## 4 Conclusion

Theories of conflict classification typically divide conflict interference effects into two components, which, from the perspective of cognitive processing, manifest as perceptual conflict and response conflict. As a non-strategic, structure-independent form of early-stage implicit conflict, perceptual conflict holds independent research value. However, it is difficult to investigate it independently within the structure of typical paradigms. Therefore, based on existing theoretical concepts and empirical evidence, this paper proposes the Task-Irrelevant Paradigm and conducts a comprehensive analysis of its theoretical definition, characteristic features, explanatory value, potential applications, and limitations.

As a novel experimental design, the Task-Irrelevant Paradigm provides an operational path for more clearly isolating perceptual conflict by decoupling task goals from conflict dimensions. It also helps clarify how conflict information may still be automatically extracted under task-irrelevant conditions, thereby highlighting its theoretical relevance to the study of unconscious processing, implicit relationships, and social metaphor. In addition, when combined with stimuli from paradigms such as Stroop, Navon, and Flanker, it shows preliminary applicability and empirical support.

However, direct research on the Task-Irrelevant Paradigm remains limited, and the results across studies vary to some extent. Therefore, more empirical support is needed for its construction principles, effect stability, and boundary conditions. Future research can begin by investigating the influencing factors of the Task-Irrelevant Paradigm, with the aim of establishing a standardized framework and advancing multimodal integration research.

## Footnotes

1 The study refers to the “Stroop paradigm” in its narrative, but an analysis of the stimulus presentation suggests that its structure aligns with a standard Navon stimulus combined with an irrelevant task design.
